# Online Community Detection for Large Complex Networks

**DOI:** 10.1371/journal.pone.0102799

**Published:** 2014-07-25

**Authors:** Gang Pan, Wangsheng Zhang, Zhaohui Wu, Shijian Li

**Affiliations:** Department of Computer Science, Zhejiang University, Hangzhou, Zhejiang, China; University of Zaragoza, Spain

## Abstract

Complex networks describe a wide range of systems in nature and society. To understand complex networks, it is crucial to investigate their community structure. In this paper, we develop an online community detection algorithm with linear time complexity for large complex networks. Our algorithm processes a network edge by edge in the order that the network is fed to the algorithm. If a new edge is added, it just updates the existing community structure in constant time, and does not need to re-compute the whole network. Therefore, it can efficiently process large networks in real time. Our algorithm optimizes expected modularity instead of modularity at each step to avoid poor performance. The experiments are carried out using 11 public data sets, and are measured by two criteria, modularity and NMI (Normalized Mutual Information). The results show that our algorithm's running time is less than the commonly used Louvain algorithm while it gives competitive performance.

## Introduction

Complex networks describe a wide range of systems in nature and society [1]–[3]. Frequently cited examples include the Internet in which routers and computers are connected by physical links, and collaboration networks in which researchers are linked by coauthoring. To understand the formation, evolution, and function of complex networks, it is crucial to investigate their community structure, not only for uncovering the relations between internal structure and functions, but also for practical applications in many disciplines such as biology and sociology [4]–[6].

Intuitively, a community of a complex network consists of a cohesive group of nodes that are relatively densely connected to each other but sparsely connected to other dense groups in the network [7]. Community detection aims to identify the communities by only using the information encoded in the network topology [8]. It is one of the critical issues in the study of complex networks. A wide variety of community detection methods have been developed to serve different scientific needs [8], [9].

Modularity is a commonly used criterion for community detection. It was first proposed in Newman *et al.* [10]. Good *et al.* [11] describe the performance of modularity maximization in practical contexts and present a broad characterization of its performance in such situations. A wide variety of algorithms for solving the modularity optimization problem have been developed [12]. For example, Clauset *et al.* [13] present a hierarchical agglomeration algorithm for detecting communities. Newman *et al.* [14] show that the modularity can be expressed in terms of the eigenvectors of a characteristic matrix for the network. This expression leads to a spectral algorithm for community detection. Modularity can be generalized in a principled fashion to incorporate the edge information such as direction and weight. Leicht *et al.* [15] consider the problem of finding communities in directed networks. Newman *et al.* [16] point out that weighted networks can, in many cases, be analyzed using a simple mapping from a weighted network to an unweighted multigraph. Lancichinetti *et al.* [9] generate directed and weighted networks with built-in community structure and show how modularity optimization performs on their benchmark. However, Fortunato *et al.* [17] find that modularity optimization may fail to identify communities smaller than a scale which depends on the total size of the network and on the degree of interconnectedness of the communities, which is called a resolution problem. To mitigate the resolution issue, Reichardt *et al.* [18] show how community detection can be interpreted as finding the ground state of an infinite range spin glass. Ruan *et al.* [19] propose a recursive algorithm HQCUT to solve the resolution limit problem. Arenas *et al.* [20] propose a method that allows for multiple resolution screening of modular structures. Aldecoa *et al.* [21] introduce a criteria called “Surprise” to resolve the resolution problem.

In some kinds of complex networks, new edges continually appear while old edges do not disappear, resulting in a large network. For example, citation networks are growing as new papers cite existing papers. To efficiently process these kinds of networks, we desire a community detection algorithm that will be able to process a network (1) without recomputing whole network after every new edge/node and (2) without the need of whole network structure available at each update. Although many community detection algorithms have been proposed, to our best knowledge, there is no algorithm that can meet these two requirements. Many existing algorithms need to start from the beginning when the network is expanded, even when only one node or edge is added.

Many efforts have been made to meet the two requirements. Leung *et al.* [22] identified novel characteristics and drawbacks of label propagation algorithm, and extended it by incorporating different heuristics to facilitate reliable and multi-functional real time community detection. Huang *et al.* [23] introduced a new quality function of local community, and presented a fast local expansion algorithm for uncovering communities in large-scale networks. Kawadia *et al.* [24] presented a new measure of partition distance called estrangement, and showed that constraining estrangement enables it to find meaningful temporal communities in diverse real-world data sets. However, both Leung's algorithm and Huang's algorithm cannot handle growing networks, since they must recompute the whole network after every new edge/node. Kawadia's algorithm requires the whole network structure to be available at each update.

In this paper, we develop a community detection algorithm to meet the two requirements. Our algorithm is an online algorithm, i.e. it can process a network edge by edge in the order that the network is fed to the algorithm, without having the whole network available from the start. Our algorithm updates existing community structure in constant time once a new edge is added. The update avoids re-processing the whole network structure, since it only needs knowledge about a network's local structure related to the new edge, thus our algorithm can efficiently process large networks in real time. Our algorithm has 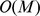 time complexity and 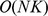 space complexity, where 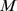 is number of edges, 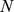 is number of nodes, and 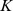 is number of communities.

This paper is an extension of our previous work [25] published in IJCAI'13 (downloaded for free in http://ijcai.org/papers13/Papers/IJCAI13-281.pdf). The main differences are three-fold: (1) This paper proposes a generative model for complex network based on preferential attachment mechanism, which helps us to infer network's future structure by its current structure and gives a solid theoretical motivation to the algorithm; (2) This paper develops a deterministic online community detection algorithm, which uses expected modularity to make an informed choice. The conference paper's non-deterministic algorithm may need many runs; (3) This paper uses additional datasets and extensive experiments for more convincing results.

## Method

To achieve the online community detection, we first propose a generative model for complex networks based on the preferential attachment mechanism [26], [27], which helps us to predict a network's future structure based on its current structure. We then develop an online community detection algorithm, which processes a network edge by edge. It optimizes expected modularity instead of modularity to avoid poor performance in some specific cases. Expected modularity can be calculated based on our generative model.

### Preliminaries

A network 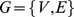 is a set of 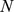 nodes 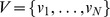 connected by a set of 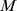 edges 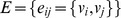. The network considered here is undirected, unweighted, and without self-loops or isolated node. Let 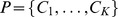 denote a partition of 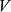. It is a division of 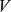 into 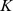 non-overlapping and non-empty communities 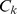 that cover all of 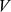. As a performance measure for the partition quality, modularity was first proposed by Newman *et al.* [28]. It can be expressed as 
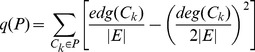
(1)where 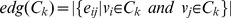 is the number of intra-community edges within community 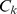, 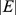 is the number of edges within network 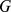, and 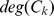 is the degree of community 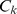, defined as 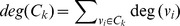, where 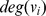 is the degree of node 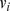. Hence community detection can be formulated as a modularity optimization problem 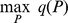
and Brandes *et al.* [29] prove the conjectured hardness of this problem both in the general case and in the case with restriction to number of partitions 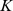. This result makes heuristic techniques the only viable option for modularity optimization problem. However, heuristic techniques cannot guarantee that the partition is good enough. It may result in a poor partition for some networks. In other words, the algorithms fail to achieve an acceptable modularity. We say an algorithm encounters failure if all nodes are assigned to the same community.

### Generative Model for Complex Network

Complex networks have non-trivial topological features that do not occur in some simple networks but often occur in real networks. An important feature of many complex networks is that their degree distributions follow a particular mathematical function called the power law [27], [30], [31], although it does not always hold [32]. The power law implies that the degree distribution of the network has no characteristic scale.

It is widely recognized as a seminal work presenting a model for the observed stationary scale-free distributions of complex networks by Price *et al.* [26]. Barabasi *et al.* [27] conclude that this feature is a consequence of two generic mechanisms: (1) networks expand continuously by the addition of new nodes; (2) new nodes attach preferentially to communities that are already well connected. Barabasi's model is recognized by academia [33], [34]. Specifically, a new node 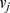 will attach to an existing node 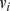 with probability 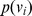 in proportion to the degree of node 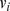


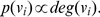
(2)


The above model only considers the case that a new edge links a new node to an existing node. However, a new edge may link two existing nodes or two new nodes. In fact, estimating the likelihood of the appearance of a new edge between two existing nodes, called *link prediction*, is one of the fundamental problems in network analysis. A variant of preferential attachment mechanism can be used to do link prediction [35]. Specifically, a new edge will link two existing nodes 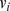 and 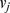 with probability 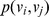 in proportion to the product of the degree of node 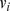 and the degree of node 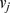


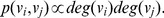
(3)


For a complete review of the statistical mechanics of network topology and dynamics of complex networks, one can refer to Boccaletti *et al.* [34] or Albert *et al.* [36]. Mitzenmacher *et al.* [37] briefly surveyed some other generative models that lead to scale-free distributions. For a summary of recent progress about link prediction algorithms, one can refer to Lu *et al.* [38].

To facilitate subsequent work, we generalize a preferential attachment mechanism from node to community. A new node will attach to an existing community 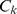 with probability 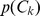 in proportion to the degree of community 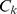


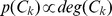
and a new edge will link two existing communities 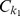 and 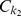 with probability 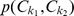 in proportion to the product of the degree of community 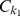 and the degree of community 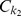


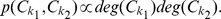



Here we propose a generative model for complex networks. Our model generates a network 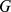 with 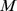 edges by addition of new edges. It is starting from an empty network 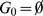. For 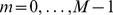, there are three cases for a new edge 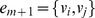 to be added in network 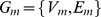, namely,

**Case (a)**: link a new node to an existing node, 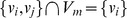 or 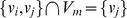, with probability 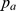;

**Case (b)**: link two existing nodes, 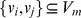, with probability 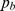;

**Case (c)**: link two new nodes, 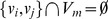, with probability 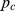.

**Figure 1:**
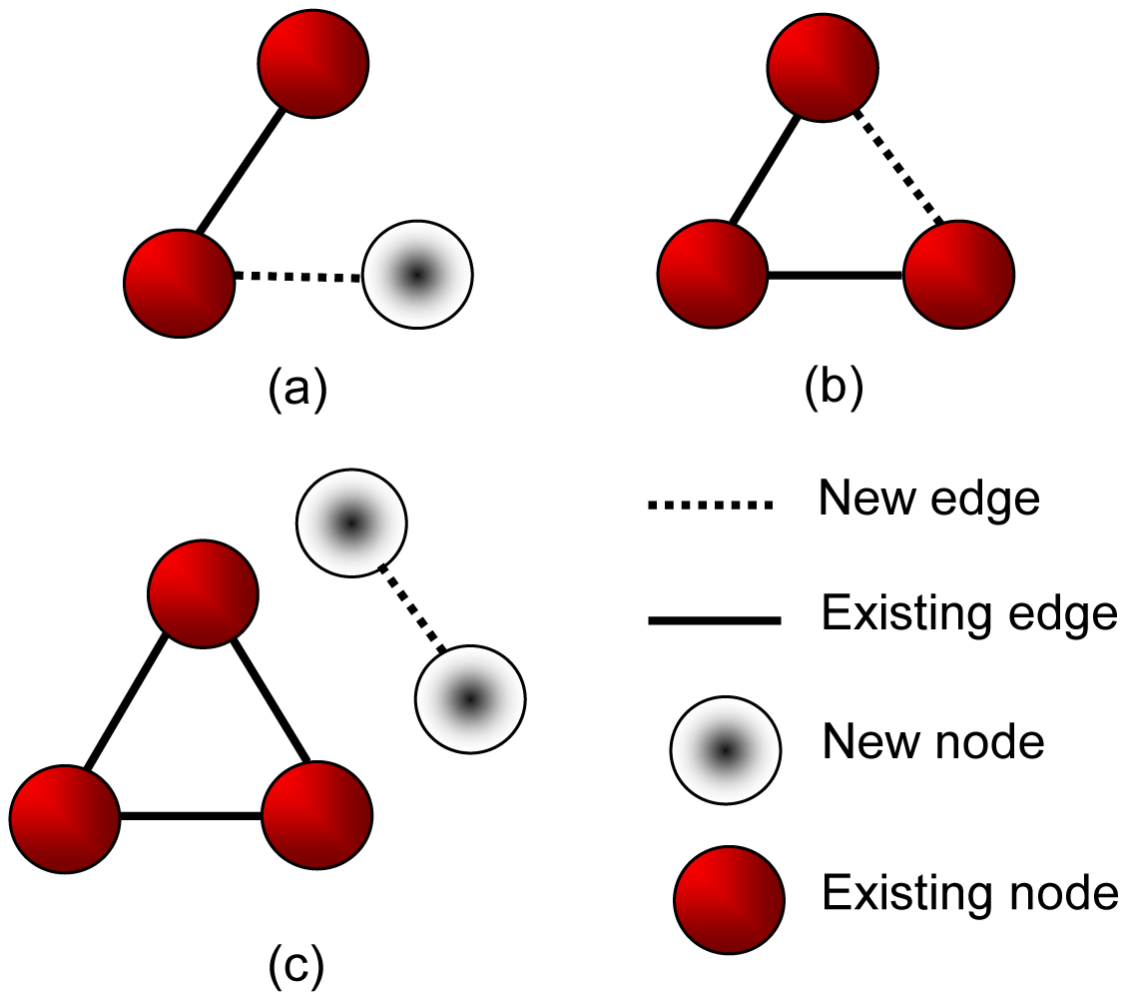
Three cases for a new edge to be added in an existing network. (a) linking a new node to an existing node; (b) linking two existing nodes; (c) linking two new nodes.

For case (a) and (b), the addition of the new edge follows preferential attachment mechanism mentioned above (See Fig. 1). Notice that 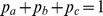. When 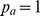, our model is the same as Barabasi's model for growing networks.

### Online Community Detection Algorithm

A straightforward way to do online community detection is to take a sequence of edges as input, and optimize modularity 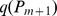 at each step for current network 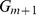 based on previous partition 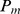. However, this greedy algorithm may have poor performance. Considering Barabasi's model that every new edge links a new node to an existing node, Brandes *et al.* [29] prove that a partition with maximum modularity has no community that consists of a single node with degree one, and a new node should be assigned to an existing community, however this operation makes all nodes in a same community and results in zero modularity.

To avoid poor performance, our algorithm optimizes expected modularity 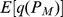 for final network 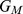, instead of modularity 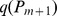 for current network 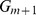 at each step. We calculate 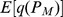 based on our generative model and the partition as follows: for existing nodes, we keep them in their current communities; for new nodes, we assign them to the corresponding existing communities to keep the degree of every existing community (defined as sum of degree of nodes which belong to that community) increasing and the expected increment of the degree of community is proportional to the degree of community. Such partition can make subsequent deriving of expected modularity simple.

First we calculate 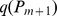. Notice that 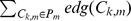 can be expressed as 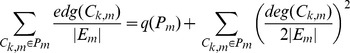
where 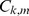 is community 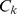 at step 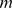 and 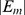 is the number of edges within network 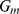, 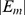 is always equal to 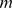 as our algorithm processes one edge at one step. Hence 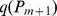 can be expressed as 
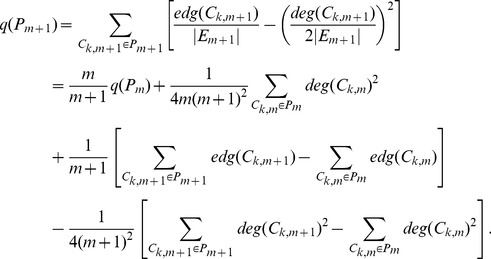
(4)


Then we calculate 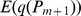 under three cases separately as follows:

**Case (a)**: link a new node to an existing node. Without loss of generality, we assume 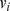 is the existing node and 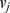 is the new node. We assign the new node to the same community as the existing node and have 
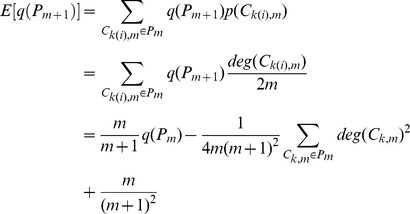
where 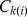 is the community which node 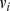 belongs to.

**Case (b)**: link two existing nodes. We do not change the partition and have 
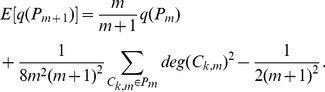



**Case (c)**: link two new nodes. We assign two new nodes to an existing community with probability in proportion to the degree of the existing community. Case (c)'s 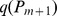 and 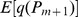 are the same as case (a)'s.

Finally we calculate 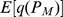 by combining 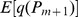 under three cases together and applying it iteratively 
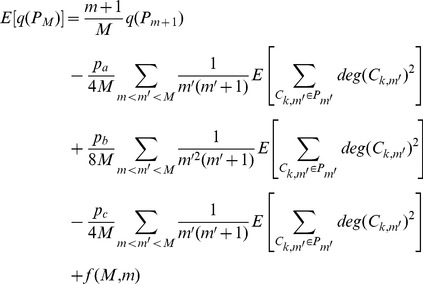
(5)where 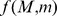 only depends on 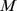 and 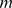.

As our partition keeps the degree of every existing community increasing, we have 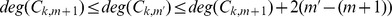
and the expected increment of the degree of community is proportional to the degree of community, thus the expected degree of community 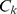 at step 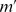 can be expressed as 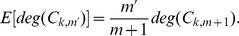



According to the Popoviciu inequality on variance, the variance of 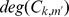 has a loose upper bound 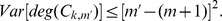



So we have 
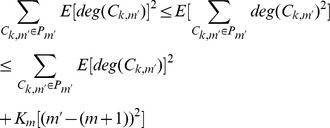
where 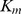 is the number of communities within network 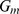 and 
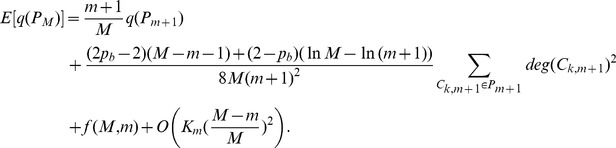
(6)


Now we describe the online community detection algorithm. For initial network 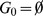, it is clear that the best partition 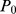 is an empty set too. For subsequent networks 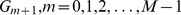, we consider some candidate operations which update the partition. Each operation has its corresponding 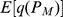. We take the operation which has the largest 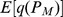. In fact, we only need to know expected modularity gain 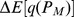, which is defined as 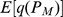 of one operation minus 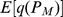 of another 
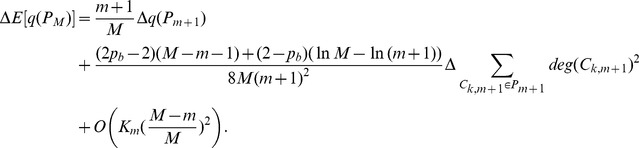
(7)


**Figure 2:**
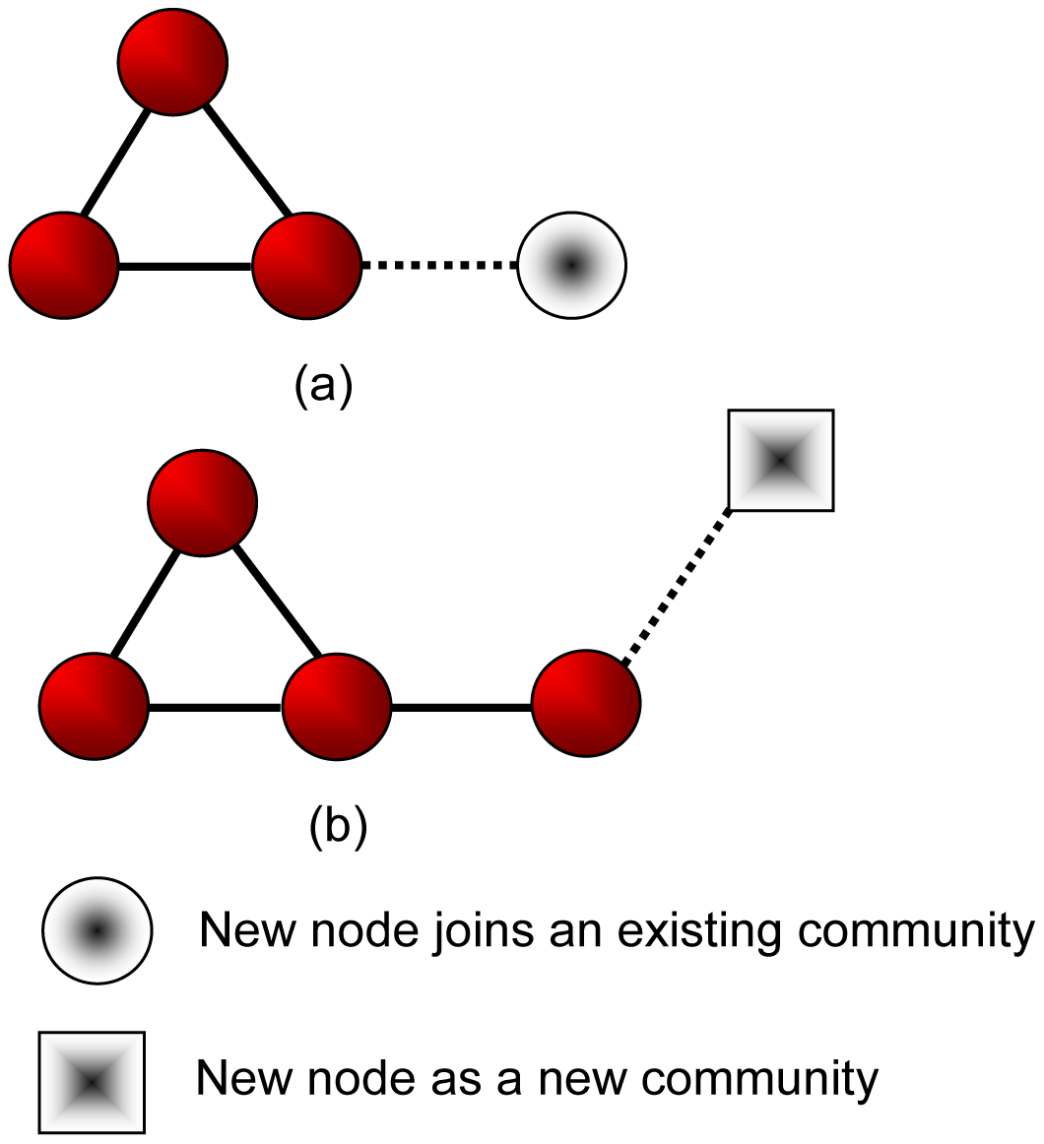
Two operations to process a new edge linking a new node to an existing node. (a) A new node attaches to an existing node with degree two, it joins the same community as the existing node; (b) Another new node attaches to the previous new node with degree one, it splits as a new community.

We describe our operations under three cases separately as follows:

**Case (a)**: link a new node to an existing node. We consider two operations: the *Split* operation where the new node splits as a new community, and the *Join* operation where the new node joins the same community as the existing node (See Fig. 2). Without loss of generality, we assume 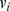 is the existing node and 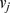 is the new node.

For the *Split* operation, we have 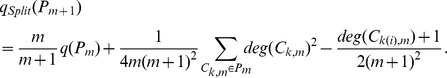



The existing community 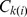 has degree 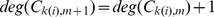 and the new community 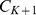 has degree 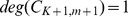 at step 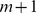.

For the *Join* operation, we have 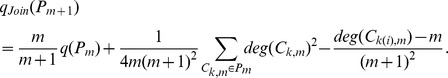



The existing community 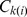 has degree 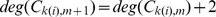 at step 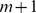.

Then we have 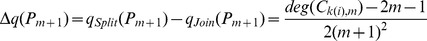
and 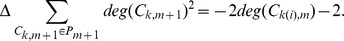



We estimate 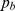 by observed frequency of case (b). Taking together and omitting the error term, we can obtain 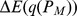, and take the *Split* operation if it is positive or the *Join* operation otherwise.

**Figure 3:**
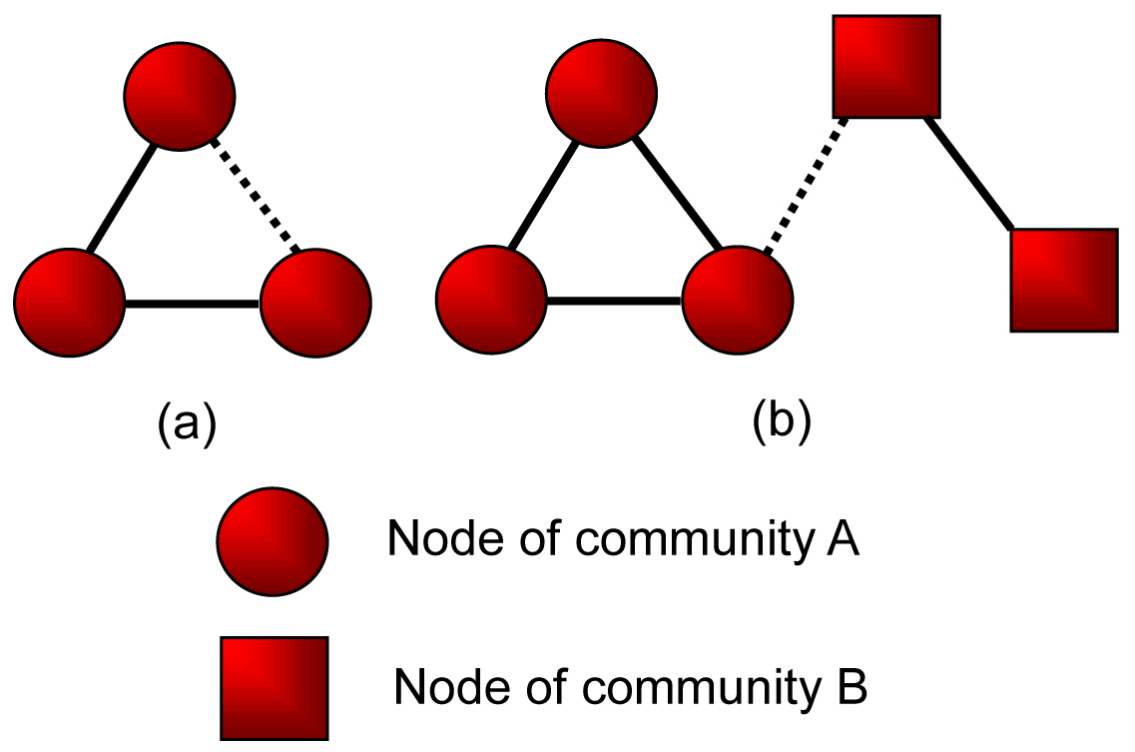
Two situations of a new edge linking two existing nodes. (a) Nodes belong to a same community; (b) Nodes belong to different communities.

**Case (b)**: link two existing nodes, two existing nodes may or may not belong to the same community (See Fig. 3). If both nodes belong to the same community, it is hard to propose a suitable candidate operation. So, we take the *Dense* operation where we keep current partition unchanged. Otherwise we consider two operations: (1) the *Move* operation where we move one node from its community to another node's community; (2) the *Keep* operation where we keep the current partition unchanged. Without loss of generality, we assume 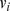 is the moving node and have 
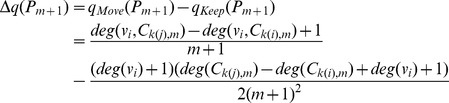
where 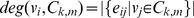 is number of edges from the node 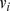 to the community 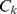 at step 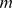 and 
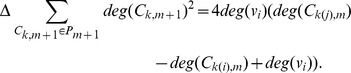



Therefore, we obtain 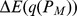 and determine the operation in the same way as we do in case (a).

**Case (c)**: link two new nodes, we consider two operations: the *New* operation where we assign two new nodes to a new community and the *Merge* operation where we assign them to an existing community. We have 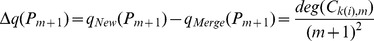
where 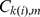 is the existing community and 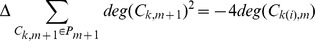



Notice that 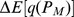 is almost always positive for large complex networks. So we take the *New* operation for case (c) to reduce complexity.

In summary, our algorithm takes a sequence of edges as input and optimizes expected modularity at each step. We assign node to community according to the maximum expected modularity gain principle. If only one node of the current edge belongs to the existing network, we split another node to a new community if this operation can maximize expected modularity gain, otherwise we let it join the same community as the existing node; if both nodes of current edge belong to the existing network but they belong to different communities, we move one node according to the same principle; if neither node of current edge belongs to the existing network, we just assign them to a new community. Obviously, our algorithm has 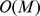 time complexity. The space complexity is 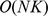 because we need to store 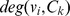 for calculating expected modularity gain in constant time. Our algorithm has two major advantages: (1) the update only uses knowledge about network's local structure related to the new edge; (2) the update can be done in constant time. Thus it can efficiently process large networks in real time.

## Results

In this section, we present the experimental results of our online community detection algorithm and compare it with a state-of-the-art algorithm, Louvain algorithm, proposed by Blondel *et al.* [39]. For simplicity, we use *OLEM* to refer to our algorithm, *OLTM* to refer to a simplified version of our algorithm which greedily optimizes temporal modularity 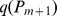 (See Eq.(4)) instead of expected modularity 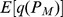 (See Eq.(6)), and *Louvain* to refer to the Louvain algorithm.

The experiments use 11 public real-world large network data sets from Stanford Large Network Dataset Collection (http://snap.stanford.edu/data/), which are commonly used by researchers. Their number of nodes varies from 12,008 to 2,394,385 and their number of edges varies from 93,439 to 4,659,565 (See Table 1). These data sets are

**Table 1:** Summary of network data sets.

Data set	Number of nodes	Number of edges
ca-CondMat	23,133	93,439
ca-HepPh	12,008	118,489
email-Enron	36,692	183,831
ca-AstroPh	18,772	198,050
cit-HepTh	27,770	352,285
cit-HepPh	34,546	420,877
com-Amazon	334,863	925,872
com-DBLP	317,080	1,049,866
web-Stanford	281,903	1,992,636
Amazon0601	403,394	2,443,408
WikiTalk	2,394,385	4,659,565

• **ca-CondMat**: Collaboration network of Arxiv Condensed Matter [40];

• **ca-HepPh**: Collaboration network of Arxiv High Energy Physics [40];

• **email-Enron**: Email communication network from Enron [41];

• **ca-AstroPh**: Collaboration network of Arxiv Astro Physics [40];

• **cit-HepTh**: Arxiv High Energy Physics paper citation network [42];

• **cit-HepPh**: Arxiv High Energy Physics paper citation network [40];

• **com-Amazon**: Amazon product network with labeled community structure [43];

• **com-DBLP**: DBLP collaboration network with labeled community structure [43];

• **web-Stanford**: Web graph of Stanford.edu [41];

• **Amazon0601**: Amazon product co-purchasing network from June 1 2003 [44];

• **WikiTalk**: Wikipedia talk (communication) network [45].

The edges should be processed in the same order as expanding procedure of the networks. However, those data sets do not have timestamps on the edges. In the experiments, we process the edges in order of their appearance in the raw files.

We use C# to implement our algorithms (Our C# implementation can be downloaded from http://www.cs.zju.edu.cn/~gpan/code/pone2013.zip). For comparison, we employ the C implementation of the Louvain algorithm provided by the authors (https://sites.google.com/site/findcommunities/). We carry out experiments on a Windows based Genuine Intel (R) CPU i7 @ 2.70 GHz machine with 4.00 GB memory.

**Figure 4:**
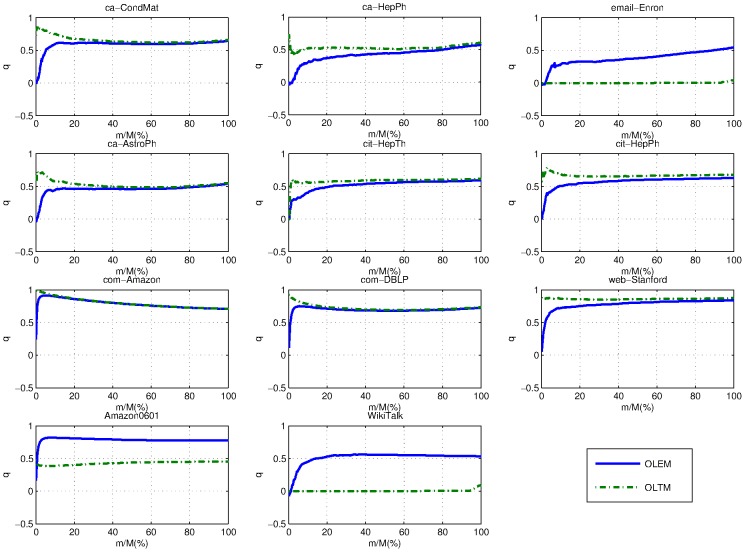
The evolution of temporal modularity over time by *OLEM* and *OLTM*.

Modularity and average running time (in seconds) over 10 runs by *OLEM*, *OLTM*, and *Louvain* are reported in Table 2 and Table 3. The evolution of temporal modularity over time by *OLEM* and *OLTM* is shown in Fig. 4.

**Table 2:** Modularity by three community detection algorithms.

Data set	*OLEM*	*OLTM*	*Louvain*
ca-CondMat	0.6446	0.6585	0.7288
ca-HepPh	0.5734	0.6052	0.6549
email-Enron	0.5447	0.0464	0.5876
ca-AstroPh	0.5418	0.5523	0.6149
cit-HepTh	0.5885	0.6146	0.6571
cit-HepPh	0.6278	0.6771	0.7228
com-Amazon	0.7050	0.7057	0.9256
com-DBLP	0.7252	0.7335	0.8091
web-Stanford	0.8377	0.8702	0.9256
Amazon0601	0.7785	0.4533	0.8670
WikiTalk	0.5344	0.0897	0.5831

**Table 3:** Average running time (in seconds) over 10 runs by three community detection algorithms.

Data set	*OLEM*	*OLTM*	*Louvain*
ca-CondMat	0.3120	0.2683	0.5029
ca-HepPh	0.3344	0.2387	0.3872
email-Enron	0.6396	0.4040	0.7004
ca-AstroPh	0.6334	0.5257	0.5806
cit-HepTh	1.0972	0.8502	1.0353
cit-HepPh	1.3736	1.1576	1.1707
com-Amazon	5.0592	4.7429	6.6453
com-DBLP	4.6914	4.3096	6.9377
web-Stanford	5.8879	5.1023	32.0137
Amazon0601	9.8601	8.1857	12.7132
WikiTalk	25.0043	17.6910	27.0956

We can see that *OLTM* is faster than *Louvain* in all data sets and *OLEM* is faster than *Louvain* in many data sets except ca-AstroPh, cit-HepTh and cit-HepPh. With the modularity measure, *OLEM* and *OLTM* cannot achieve similar performance to *Louvain*. This is due to our algorithms being online one-pass algorithms while *Louvain* is an offline multi-pass algorithm. Our algorithms' running times are linear in number of edges as we expected while *Louvain* is not. This is due to the number of passes of *Louvain* is not fixed. Most of all, *Louvain* needs to start from the beginning when a new edge is added while our algorithms do not.

*OLTM* is faster than *OLEM* because 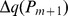 is simpler than 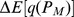. In fact, we calculate 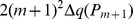 instead of 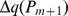 in our implementation as the former only involves integer arithmetic which is faster than float-point arithmetic. *OLEM* keeps relatively stable performance in all data sets while *OLTM* has exceptionally poor performance in the email-Enron and WikiTalk data sets. We will further investigate the underlying cause for *OLTM* later. *OLTM* often performs slightly better than *OLEM* in the other data sets. It may be due to our approximation of expected modularity by a lower bound in *OLEM*.

As we mentioned in the Introduction Section, the modularity optimization based approach may fail to identify communities smaller than a scale, which is called a resolution limit problem [17]. To investigate this problem, we compare results of *OLEM*, *OLTM* and *Louvain* in the com-Amazon and com-DBLP data sets. We choose the two data sets because Yang *et al.* [43] released a labeled community structure for either of the data sets (http://snap.stanford.edu/data/com-Amazon.html, http://snap.stanford.edu/data/com-DBLP.html). For com-Amazon data set, Yang *et al.* labeled products from the same category as a community and nodes (products) that belong to a common community share a common function or purpose. For com-DBLP data set, they labeled authors who published to a certain journal or conference as a community and nodes (authors) that belong to a common community share a comon research interest. For each data set, we use the top 5,000 subset, same as [43], for comparison.

We find that, although both our method and the *Louvain* method optimize the modularity function, the number of communities in *Louvain*'s result is less than that in our results (See Table 4). It is due to our method and the *Louvain* method achieving optimization in different ways. The *Louvain* method optimizes the modularity function by merging pair of communities in each pass, while our method optimizes the modularity function by moving nodes of the new edge at each step in order to satisfy the real-time processing. Generally speaking, merging communities may obtain higher modularity gain than moving nodes, so the *Louvain* method is better than our method to optimize the modularity. However, merging communities in each pass will reduce the number of communities in final result as each merging operation will eliminate one community. It causes that the *Louvain* method will miss small communities.

**Table 4:** Number of communities by three community detection algorithms and Yang's labeled community structure.

Data set	*OLEM*	*OLTM*	*Louvain*	*Labeled*
Amazon	1,988	1,979	217	5,000
DBLP	4,122	3,854	301	5,000

Further, the similarity between the results and labeled community structures can be measured by NMI (Normalized Mutual Information) criterion [46]. We find that, measured in NMI, our results are more similar to labeled community structure than *Louvain*'s result (See Table 5). The main reason may be that our methods can find more communities of small scale, which the *Louvain* method may be hard to identify.

**Table 5:** NMI Benchmark by three community detection algorithms comparing with Yang's labeled community structure.

Data set	*OLEM*	*OLTM*	*Louvain*
Amazon	0.7261	0.7273	0.3118
DBLP	0.2355	0.2376	0.1958

The reason for *OLTM*'s poor performance in the email-Enron and WikiTalk data sets is that *OLTM* has no *Split* operation for case (b) edge. As *OLTM* is a greedy approach, it only takes the *Join* operation for case (b) edge to maximize temporal modularity. Hence the only way for *OLTM* to create new community is its *New* operation for case (c) edge. If a data set has few case (c) edges at its beginning, *OLTM* cannot create enough communities in the early stage and obtains a poor final partition. In the worst situation, the data set has no case (c) edge and *OLTM* fails. In fact, email-Enron and WikiTalk data sets have very few case (c) edges at their beginning, comparing with the other data sets.

In contrast, with the help of expected modularity, *OLEM* can take the *Split* operation for case (b) edge. Hence it can create enough communities in the early stage and obtains an acceptable final partition in email-Enron and WikiTalk data sets.

**Figure 5:**
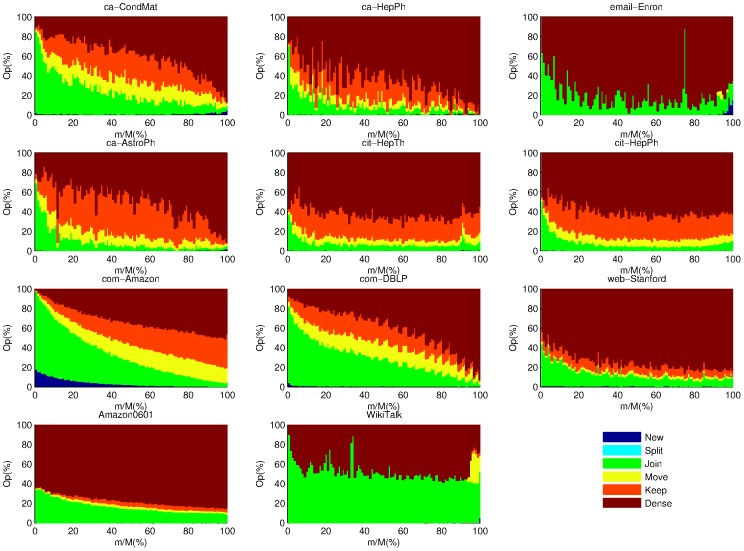
The percentage of different operations of *OLTM* over time. The height of each color segment represents the percentage of an operation at a certain progress. “Op” is the abbreviation for “Operation” of *OLTM* at each step.

**Figure 6:**
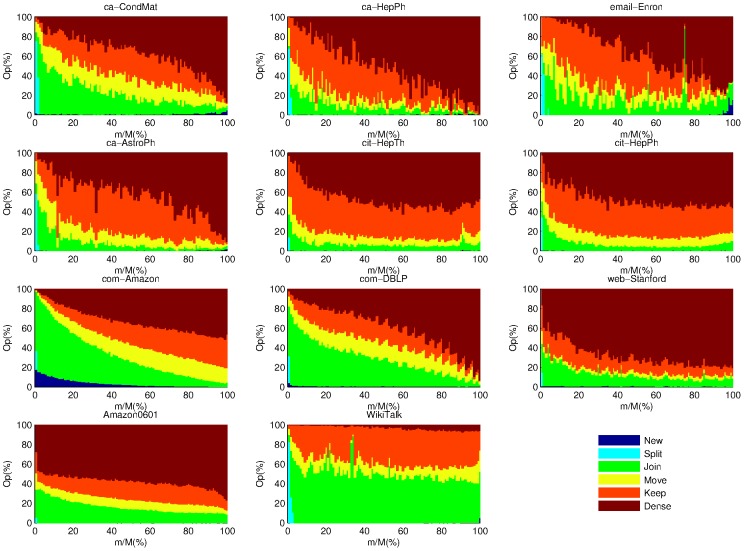
The percentage of different operations of *OLEM* over time. The height of each color segment represents the percentage of an operation at a certain progress. “Op” is the abbreviation for “Operation” of *OLEM* at each step.

To compare *OLTM* and *OLEM*'s operations, we plot the percentage of different operations of *OLTM* and *OLEM* over time in Fig. 5 and 6. We can see that *OLTM* generally only takes the *Join* and *Dense* operations until very later stage while *OLEM* takes many *Split* operations in the early stage in the email-Enron and WikiTalk data sets. Therefore, *OLEM*'s temporal modularity increases steadily over time while *OLTM*'s temporal modularity remains zero until very later stages in email-Enron and WikiTalk data sets (See Fig. 4). In fact, *OLEM* can obtain an acceptable modularity even in early stage for the email-Enron and WikiTalk data sets.

As a statistical analysis, we created 10 copies of each original data set with the edges randomly reordered and ran our algorithm on those reordered data sets. The statistics of modularity in those reordered data sets as well as modularity in original data set are reported in Table 6. Modularity in original data set is significantly better than those in reordered data sets for ca-CondMat, email-Enron, com-Amazon, com-DBLP, web-Stanford, and Amazon0601. We guess, for these six data sets, the storing order of edges may be close to the order of their expanding. For the other data sets, modularity difference between the original and reordered is slight. We think the edges may be not stored by their creation time in those data sets.

**Table 6:** The statistics of modularity on 10 reordered data sets as well as modularity on original data set by our algorithm.

Data set	q(Original)	AVG(q)	MAX(q)	MIN(q)
ca-CondMat	0.6446	0.5344	0.5375	0.5298
ca-HepPh	0.5734	0.5844	0.5872	0.5823
email-Enron	0.5447	0.4730	0.4872	0.4541
ca-AstroPh	0.5418	0.5468	0.5536	0.5427
cit-HepTh	0.5885	0.5777	0.5873	0.5588
cit-HepPh	0.6278	0.6376	0.6524	0.6288
com-Amazon	0.7050	0.5903	0.5916	0.5895
com-DBLP	0.7252	0.5706	0.5715	0.5694
web-Stanford	0.8377	0.7431	0.7501	0.7385
Amazon0601	0.7785	0.5682	0.5708	0.5647
WikiTalk	0.5344	0.5102	0.5104	0.5101

## Conclusions

In this paper we have examined the problem of online community detection for large complex networks in which new edges continually appear while old edges do not disappear. We have formulated it as a modularity optimization problem. We have proposed a generative model for complex networks and developed an online algorithm with linear time complexity. Our algorithm processes a network edge by edge in the order that the network is fed to the algorithm. It does not optimize modularity but expected modularity to avoid poor performance. The two major advantages of our algorithm are (1) the update only uses knowledge about network's local structure related to the new edge; (2) the update can be done in constant time. Our algorithm can efficiently process large networks in real time. The algorithm has been applied to 11 public real-world large network data sets and our experiments give very encouraging results. Not only is the proposed algorithm scalable in terms of both time and space complexity, but it also gives comparable performance. Our future research will consider (1) combining *OLTM* and *OLEM* into a better one; (2) improving the generative model to allow edge to appear and disappear in general probability distribution; (3) exploring how to apply our method to other objective functions.

## References

[1] Rubinov M, Sporns O (2010) Complex network measures of brain connectivity: Uses and interpretations. NeuroImage 52: 1059–1069. 19819337 10.1016/j.neuroimage.2009.10.003

[2] Barrat A, Barthelemy M, Pastor-Satorras R, Vespignani A (2004) The architecture of complex weighted networks. Proceedings of the National Academy of Sciences 101: 3747–3752. 10.1073/pnas.0400087101 PMC374315 15007165

[3] Phithakkitnukoon S, Calabrese F, Smoreda Z, Ratti C (2011) Out of sight out of mind–how our mobile social network changes during migration. In: Proceedings of the Third International Conference on Social Computing. pp. 515–520.

[4] Fan M, Wong KC, Ryu T, Ravasi T, Gao X (2012) Secom: A novel hash seed and community detection based-approach for genome-scale protein domain identification. PLoS ONE 7: e39475. 22761802 10.1371/journal.pone.0039475 PMC3386278

[5] Ratti C, Sobolevsky S, Calabrese F, Andris C, Reades J, et al (2010) Redrawing the map of great britain from a network of human interactions. PLoS One 5: e14248. 21170390 10.1371/journal.pone.0014248 PMC2999538

[6] Tang C, Li X, Cao L, Zhan J (2012) The law of evolutionary dynamics in community-structured population. Journal of Theoretical Biology 306: 1–6. 22554982 10.1016/j.jtbi.2012.04.024

[7] Porter MA, Onnela JP, Mucha PJ (2009) Communities in networks. Notices of the American Mathematical Society 56: 1082–1097.

[8] Fortunato S (2010) Community detection in graphs. Physics Reports 486: 75–174.

[9] Lancichinetti A, Fortunato S (2009) Community detection algorithms: a comparative analysis. Physical Review E 80: 056117. 10.1103/PhysRevE.80.056117 20365053

[10] Newman MEJ, Girvan M (2004) Finding and evaluating community structure in networks. Physical Review E 69: 026113. 10.1103/PhysRevE.69.026113 14995526

[11] Good BH, de Montjoye YA, Clauset A (2010) Performance of modularity maximization in practical contexts. Physical Review E 81: 046106. 10.1103/PhysRevE.81.046106 20481785

[12] Leskovec J, Lang KJ, Mahoney M (2010) Empirical comparison of algorithms for network community detection. In: Proceedings of the Nineteenth International Conference on World Wide Web. pp. 631–640.

[13] Clauset A, Newman MEJ, Moore C (2004) Finding community structure in very large networks. Physical Review E 70: 066111. 10.1103/PhysRevE.70.066111 15697438

[14] Newman MEJ (2006) Modularity and community structure in networks. Proceedings of the National Academy of Sciences 103: 8577–8582. 10.1073/pnas.0601602103 PMC1482622 16723398

[15] Leicht EA, Newman MEJ (2008) Community structure in directed networks. Physical Review Letters 100: 118703. 18517839 10.1103/PhysRevLett.100.118703

[16] Newman MEJ (2004) Analysis of weighted networks. Physical Review E 70: 056131. 10.1103/PhysRevE.70.056131 15600716

[17] Fortunato S, Barthelemy M (2007) Resolution limit in community detection. Proceedings of the National Academy of Sciences 104: 36–41. 10.1073/pnas.0605965104 PMC1765466 17190818

[18] Reichardt J, Bornholdt S (2006) Statistical mechanics of community detection. Physical Review E 74: 016110. 10.1103/PhysRevE.74.016110 16907154

[19] Ruan J, Zhang W (2008) Identifying network communities with a high resolution. Physical Review E 77: 016104. 10.1103/PhysRevE.77.016104 18351912

[20] Arenas A, Fernndez A, Gmez S (2008) Analysis of the structure of complex networks at different resolution levels. New Journal of Physics 10: 053039.

[21] Aldecoa R, Marn I (2011) Deciphering network community structure by surprise. PLoS ONE 6: e24195. 21909420 10.1371/journal.pone.0024195 PMC3164713

[22] Leung IXY, Hui P, Lio P, Crowcroft J (2009) Towards real-time community detection in large networks. Physical Review E 79: 066107. 10.1103/PhysRevE.79.066107 19658564

[23] Huang J, Sun H, Liu Y, Song Q, Weninger T (2011) Towards online multiresolution community detection in large-scale networks. PLoS ONE 6: e23829. 21887325 10.1371/journal.pone.0023829 PMC3161084

[24] Kawadia V, Sreenivasan S (2012) Sequential detection of temporal communities by estrangement confinement. Scientific Reports 2.. 10.1038/srep00794 PMC3494021 23145317

[25] Zhang W, Pan G, Wu Z, Li S (2013) Online community detection for large complex networks. In: Proceedings of the Twenty-Third International Joint Conference on Artificial Intelligence. pp. 1903–1909.

[26] Price DDS (1976) A general theory of bibliometric and other cumulative advantage processes. Journal of the American Society for Information Science 27: 292–306.

[27] Barabási AL, Albert R (1999) Emergence of scaling in random networks. Science 286: 509–512. 10521342 10.1126/science.286.5439.509

[28] Newman MEJ (2004) Detecting community structure in networks. The European Physical Journal B-Condensed Matter and Complex Systems 38: 321–330.

[29] Brandes U, Delling D, Gaertler M, Gorke R, Hoefer M, et al (2008) On modularity clustering. IEEE Transactions on Knowledge and Data Engineering 20: 172–188.

[30] Clauset A, Shalizi C, Newman M (2009) Power-law distributions in empirical data. SIAM Review 51: 661–703.

[31] Faloutsos M, Faloutsos P, Faloutsos C (1999) On power-law relationships of the internet topology. SIGCOMM Computer Communication Review 29: 251–262.

[32] Stumpf MPH, Porter MA (2012) Critical truths about power laws. Science 335: 665–666. 22323807 10.1126/science.1216142

[33] Newman MEJ (2003) The structure and function of complex networks. SIAM Review 45: 167–256.

[34] Boccaletti S, Latora V, Moreno Y, Chavez M, Hwang DU (2006) Complex networks: structure and dynamics. Physics Reports 424: 175–308.

[35] Xie YB, Zhou T, Wang BH (2008) Scale-free networks without growth. Physica A: Statistical Mechanics and its Applications 387: 1683–1688.

[36] Albert R, Barabási AL (2002) Statistical mechanics of complex networks. Reviews of Modern Physics 74: 47.

[37] Mitzenmacher M (2004) A brief history of generative models for power law and lognormal distributions. Internet Mathematics 1: 226–251.

[38] Lü L, Zhou T (2011) Link prediction in complex networks: A survey. Physica A: Statistical Mechanics and its Applications 390: 1150–1170.

[39] Blondel VD, Guillaume JL, Lambiotte R, Lefebvre E (2008) Fast unfolding of communities in large networks. Journal of Statistical Mechanics: Theory and Experiment 2008: P10008.

[40] Leskovec J, Kleinberg J, Faloutsos C (2007) Graph evolution: densification and shrinking diameters. ACM Transactions on Knowledge Discovery from Data 1: 2.

[41] Leskovec J, Lang KJ, Dasgupta A, Mahoney MW (2009) Community structure in large networks: Natural cluster sizes and the absence of large well-defined clusters. Internet Mathematics 6: 29–123.

[42] Leskovec J, Kleinberg J, Faloutsos C (2005) Graphs over time: densification laws, shrinking diameters and possible explanations. In: Proceedings of the Eleventh International Conference on Knowledge Discovery in Data Mining. pp. 177–187.

[43] Yang J, Leskovec J (2012) Defining and evaluating network communities based on ground-truth. In: Proceedings of the Twelfth International Conference on Data Mining. pp. 745–754.

[44] Leskovec J, Adamic LA, Huberman BA (2007) The dynamics of viral marketing. ACM Transactions on the Web 1: 5.

[45] Leskovec J, Huttenlocher D, Kleinberg J (2010) Predicting positive and negative links in online social networks. In: Proceedings of the Nineteenth International Conference on World Wide Web. pp. 641–650.

[46] Lancichinetti A, Fortunato S, Kertsz J (2009) Detecting the overlapping and hierarchical community structure in complex networks. New Journal of Physics 11: 033015.

